# GSK-3β Is Required for Memory Reconsolidation in Adult Brain

**DOI:** 10.1371/journal.pone.0003540

**Published:** 2008-10-28

**Authors:** Tetsuya Kimura, Shunji Yamashita, Shinobu Nakao, Jung-Mi Park, Miyuki Murayama, Tatsuya Mizoroki, Yuji Yoshiike, Naruhiko Sahara, Akihiko Takashima

**Affiliations:** Lab for Alzheimer's Disease, RIKEN Brain Science Institute, Wako, Saitama, Japan; Centre de Recherches su la Cognition Animale-Centre National de la Recherche Scientifique and Université Paul Sabatier, France

## Abstract

Activation of GSK-3β is presumed to be involved in various neurodegenerative diseases, including Alzheimer's disease (AD), which is characterized by memory disturbances during early stages of the disease. The normal function of GSK-3β in adult brain is not well understood. Here, we analyzed the ability of heterozygote GSK-3β knockout (GSK+/−) mice to form memories. In the Morris water maze (MWM), learning and memory performance of GSK+/− mice was no different from that of wild-type (WT) mice for the first 3 days of training. With continued learning on subsequent days, however, retrograde amnesia was induced in GSK+/− mice, suggesting that GSK+/− mice might be impaired in their ability to form long-term memories. In contextual fear conditioning (CFC), context memory was normally consolidated in GSK+/− mice, but once the original memory was reactivated, they showed reduced freezing, suggesting that GSK+/− mice had impaired memory reconsolidation. Biochemical analysis showed that GSK-3β was activated after memory reactivation in WT mice. Intraperitoneal injection of a GSK-3 inhibitor before memory reactivation impaired memory reconsolidation in WT mice. These results suggest that memory reconsolidation requires activation of GSK-3β in the adult brain.

## Introduction

Two GSK-3 isoforms (α and β) are encoded by different genes in mammalian tissues [1]. GSK-3β is highly enriched in the brain, where it phosphorylates metabolic enzymes, signal proteins, structural proteins, and transcription factors [2]–[4]. GSK-3β is a constitutively active kinase. Most GSK-3β substrates are under negative regulation, which is relieved by Ser9 phosphorylation through other kinases such as PKC, PKA, and Akt [3]. GSK-3 inhibitor experiments suggest that GSK-3 may be involved in mood disorders, schizophrenia, and pathogenesis of neurofibrillary tangles (NFTs) [4]–[7].

NFTs, neuropathological hallmarks of neurodegenerative disorders, are composed of highly phosphorylated tau [8]–[10]. GSK-3β is an enzyme potentially involved in forming highly phosphorylated tau in NFTs [11]–[14]. This hypothesis is supported by the observation that a GSK-3 inhibitor prevents NFT formation in an animal model [4], [15]. Aggregated Aβ activates GSK-3β and induces hyperphosphorylation of tau and neuronal death [14], [16], [17]. Furthermore, the overexpression of GSK-3β affects spatial memory and accelerates NFT formation in a mouse model [18], [19]. This accumulating evidence suggests that activation of GSK-3β may be involved in NFT formation and neuronal loss in neurodegenerative diseases, including Alzheimer's disease (AD).

One hypothesis explaining the role of GSK-3β in the adult brain posits that under normal conditions, GSK-3β is inhibited and does not affect brain function. However, in disease states, GSK-3β becomes activated through the elimination of inactivation signals, leading to neurodegeneration. This hypothesis provides the basis for the development of GSK-3β inhibitors as potential therapeutic drugs for treating AD and other neurodegenerative diseases. However, it is inconceivable that a constitutively active kinase such as GSK-3β exists for the purpose of neurodegeneration.

Recently, Peineau et al. [20] reported the importance of GSK-3β activity during the induction of long term depression (LTD), suggesting that GSK-3β activity may contribute to the control of synaptic plasticity and memory function. If GSK-3β is involved only in neurodegeneration, then a genetic reduction of GSK-3β would not be expected to affect cognitive function. On the other hand, if GSK-3β is involved in normal brain function, a genetic reduction of GSK-3β would be expected to affect cognitive function. To test this hypothesis, we compared memory formation and maintenance of heterozygous GSK-3β gene-deficient mice (GSK^+/−^) and their wild-type (WT) littermates using the Morris water maze (MWM) test and a contextual fear-conditioning (CFC) test. GSK^+/−^ mice lacked the ability to maintain memory after recall, suggesting GSK-3β importantly contributes to memory maintenance after recall in normal brains.

## Results

### Generation and characterization of GSK^+/−^

To understand the normal role of GSK-3β in the adult brain, we generated GSK-3β gene-deficient mice according to published methods [21] and analyzed heterozygous GSK-3β gene-deficient (GSK^+/−^) mice. The total amount of GSK-3β in GSK^+/−^ mice was approximately 50% of that in WT littermate mice, whereas the relative activity of GSK-3β in GSK^+/−^ mice was about 70% of that in WT littermates (see Supporting information Fig. S1). As previously reported [21], GSK^+/−^ mice were healthy, fertile, had normal circadian rhythms and life span, but had unimpaired motor and locomotor activity compared to their WT littermates (see Supporting information Fig. S2). This was the case even though they had reduced GSK-3β activity and lacked GSK-3α compensation (see Supporting information Fig. S1).

### GSK^+/−^ mice exhibited retrograde amnesia with repeated training in MWM

**Figure 1:**
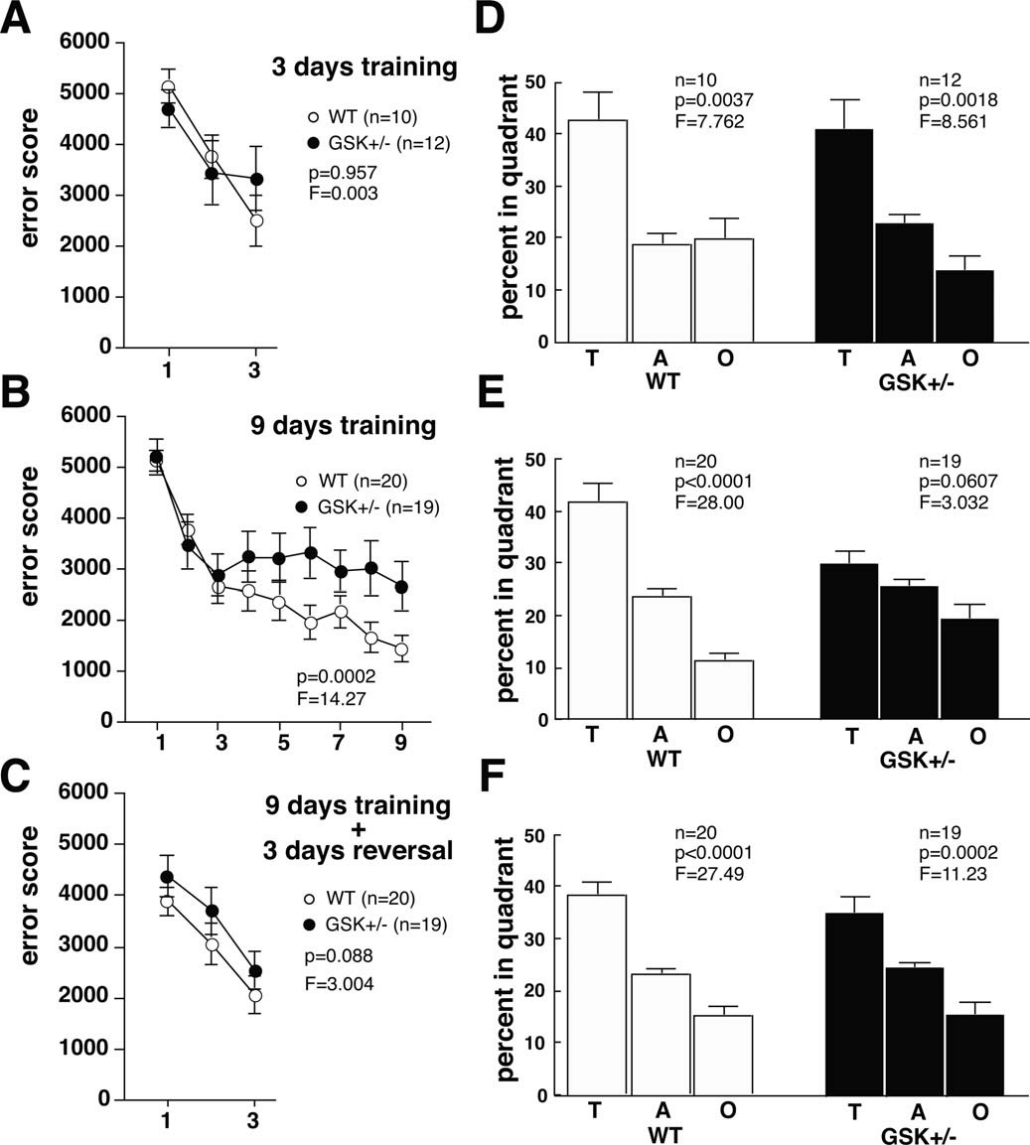
Analysis of place learning and memory in GSK-3β heterozygous mice. The Morris water maze (MWM) was used to assess place learning and memory of adult WT littermate and GSK^+/−^ mice (7–14 months old). The cognitive function of 7- to 14-month-old mice did not show age-dependent decline (Supporting information Fig. S5). Memory was assessed after 3 days of continuous training (A, D); 9 days of continuous training (B, E); and 3 days of reversal testing following the 9-day continuous training session (C, F). Learning is expressed as error scores (A, B, C), and memory performance is expressed as a percentage of total time spent in the target quadrant during the probe test (D, E, F). The probe test was given on the fourth day (D, F) or tenth day (E) of the experiment (see Methods). Differences between genotypes were statistically analyzed with a two-way ANOVA (A–C). Dwell times of GSK^+/−^ and WT littermate mice spent in the target (T), adjacent (A), and opposite (O) quadrants were statistically analyzed by one-way ANOVA (d–f). Results are expressed as means±SEM.

Adult mice (7–14 months old) were trained 3 times a day to find a fixed but hidden platform submerged in a circular pool; training occurred for 3 or 9 days. Memory performance was measured in terms of an error score, which represents the cumulative distance a mouse traveled in each trial as it searched to find the hidden platform. The error score more accurately and sensitively reflects a mouse's place preference in a trial than other parameters (Supporting information Fig. S3). Fig. 1A, B show the average error scores of the mice during the training. During the first 3 days of training, GSK^+/−^ mice learned to navigate to the hidden platform and had low error scores comparable to those of WT littermate mice (Fig. 1A). In the probe test, which was conducted after 3 days of continuous training, GSK^+/−^ mice recognized the place where the platform used to be as well as the WT mice (Fig. 1D). However, with more than 3 days of continuous training (9 days of training), GSK^+/−^ mice took longer than WT mice to locate the hidden platform. Their error scores increased, while the error scores of WT mice decreased steadily, indicating that the WT mice had effectively learned the location of the platform during the additional training days (Fig. 1B). To evaluate the spatial memory of GSK^+/−^ mice, we conducted a probe test 24 h after the last session on the ninth training day. In the probe test, GSK^+/−^ mice did not show statistically significant place preference (Fig. 1E). However, GSK^+/−^ mice that received 3 days of training (Fig. 1E) and WT mice that received 9 days of training (Fig. 1F) did show significant place preference. Thus, GSK^+/−^ mice failed to locate the previous location of the hidden platform, while WT mice located the platform easily, indicating that WT mice had retained place recognition, whereas GSK^+/−^ mice did not (Fig. 1E).

After 9 days of training, we moved the hidden platform to a quadrant opposite to that of the previous training session and continued training the mice. As with the initial test (Fig. 1A), GSK^+/−^ mice learned the place of the relocated platform during the first 3 days of training (Fig. 1C), showing comparable spatial memory to that of their WT littermates (Fig. 1F). The swimming speed (motor control ability) and thigmotaxic tendency (emotional control ability) of GSK^+/−^ and WT littermate mice on the first day of training were not significantly different (Supporting information Fig. S4), suggesting that motor and emotional factors could not account for group differences in MWM performance. The relatively wide range of ages of the mice (7–14 months) could not account for differences in cognitive ability of WT and GSK^+/−^ mice (Supporting information Fig. S5). Therefore, these results were due to genetic differences between the mice, and suggest that, although they retained the ability to learn, GSK^+/−^ mice exhibited retrograde amnesia with repeated training. Thus, GSK-3β reduction does not affect the learning process but does affect the memory maintenance system, such as memory consolidation and/or reconsolidation.

### GSK^+/−^ impairs memory reconsolidation

**Figure 2:**
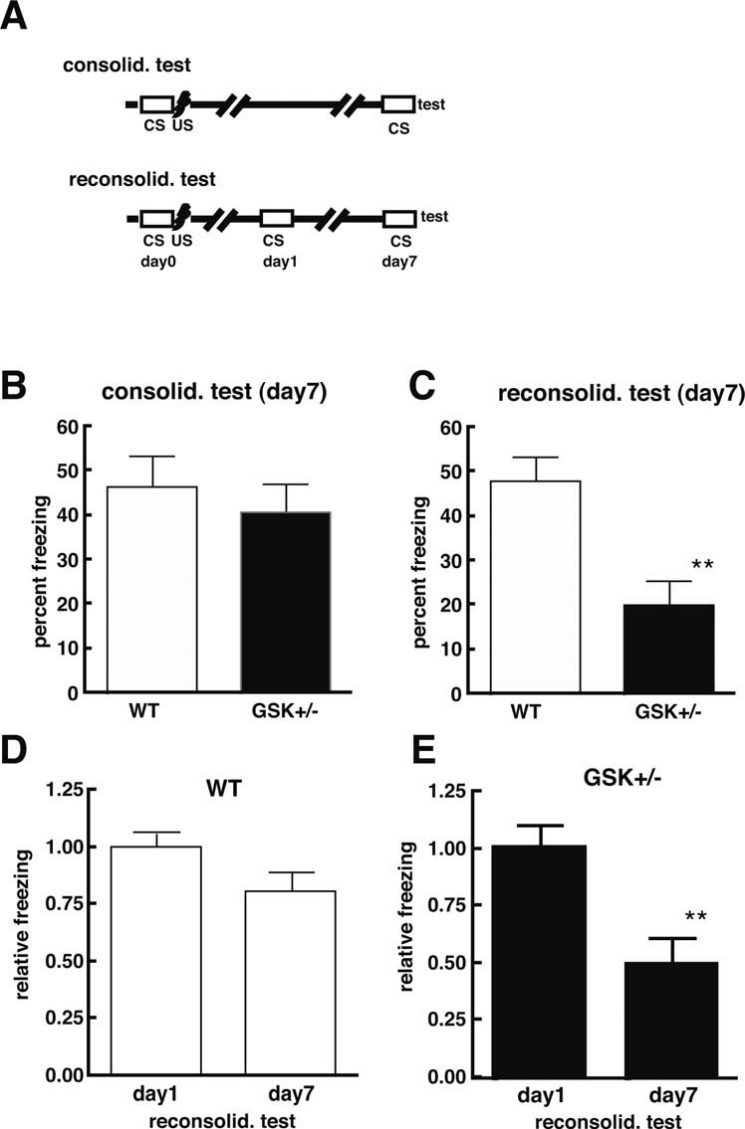
Memory consolidation and reconsolidation in GSK-3β heterozygous mice. Memory consolidation and reconsolidation were assessed with a contextual fear-conditioning (CFC) test following the schedule shown in A. Mice were placed in a novel environment (plastic chamber) for 5 min (conditioning stimulus, CS), where they received three electrical foot shocks (unconditioning stimulus, US; shock intensity = 0.5 mV; duration = 2 sec; inter-stimuli interval = 60 sec). In the consolidation test performed 7 days later, the mice (WT littermates, n = 13; GSK^+/−^, n = 14; 7–14 months old) were placed into the plastic chamber again for 5 min without receiving stimulation, and freezing time was measured (A, B). In the reconsolidation test performed on day 1 and day 7 of training, the mice (WT littermates, n = 17; GSK^+/−^, n = 11) were placed into the plastic chamber again for 5 min without receiving stimulation, and freezing time was measured (A, C–E). On day 7 of the consolidation test (B), the freezing times of WT littermate (white bar) and GSK^+/−^ (black bar) mice were not significantly different (p = 0.437, Mann-Whitney test). However, on day 7 of the reconsolidation test, the freezing times of GSK^+/−^ mice had decreased significantly compared to that of their WT littermates (c; p = 0.0026, Mann-Whitney test). Comparison of day 1 and day 7 freezing times of WT littermate (D) and GSK^+/−^ (E) mice revealed that, on day 7 of the reconsolidation test, GSK^+/−^ mice showed significantly reduced freezing times (p = 0.0095, Mann-Whitney test), while WT littermate mice did not (p>0.0733, Mann-Whitney test). Results are expressed as means±SEM; **, p<0.01.

A new memory is initially labile and becomes stabilized over time through the process of consolidation. Once stabilized, memory is not permanently fixed and can again become labile if reactivated by recall, which is called reconsolidation. The reconsolidation process is hypothesized to be necessary for updating reactivated memories [22]. Although memory consolidation and reconsolidation are thought to have distinct molecular requirements, both require a protein synthesis-dependent process [24]–[29]. To understand in which process GSK-3β is involved, we examined GSK^+/−^ mice in a CFC paradigm. GSK^+/−^ and WT littermate mice showed similar freezing times in response to the unconditioning stimulus (US). Twenty-four hours after exposure to the US, mice in the reconsolidation test group were placed in the same context (conditioning stimulus; CS) again without the US to reactivate their memory by recall. Mice in the consolidation group did not receive memory reactivation until the test phase. On day 7, memory performance of both the consolidation and reconsolidation test groups (Fig. 2A) was examined by measuring freezing time. In the consolidation test, GSK^+/−^ and WT littermate mice showed the same level of freezing (Fig. 2B), suggesting that GSK^+/−^ mice are not impaired in the ability to form and consolidate memory. Furthermore, the consolidated memory is maintained for at least 7 days. In contrast, GSK^+/−^ mice showed significantly less freezing in the reconsolidation test on day 7 compared to WT littermate mice (Fig. 2C). Comparison of day 1 and day 7 freezing times in the reconsolidation test (Fig. 2A) revealed that the freezing times of GSK^+/−^ mice decreased significantly from day 1 to day 7 (Fig. 2E), while those of WT littermate mice remained more or less unchanged (Fig. 2D). These results indicate that GSK^+/−^ mice learned and that their memory stabilized over 7 days if memory was not reactivated. However, GSK^+/−^ mice failed to achieve reconsolidation when the memory was reactivated once before testing. Therefore, GSK^+/−^ mice show retrograde amnesia in the CFC reconsolidation test as well as in the MWM test, suggesting that GSK^+/−^ mice may have impaired memory reconsolidation.

### Memory reconsolidation requires the activation of GSK-3β

**Figure 3:**
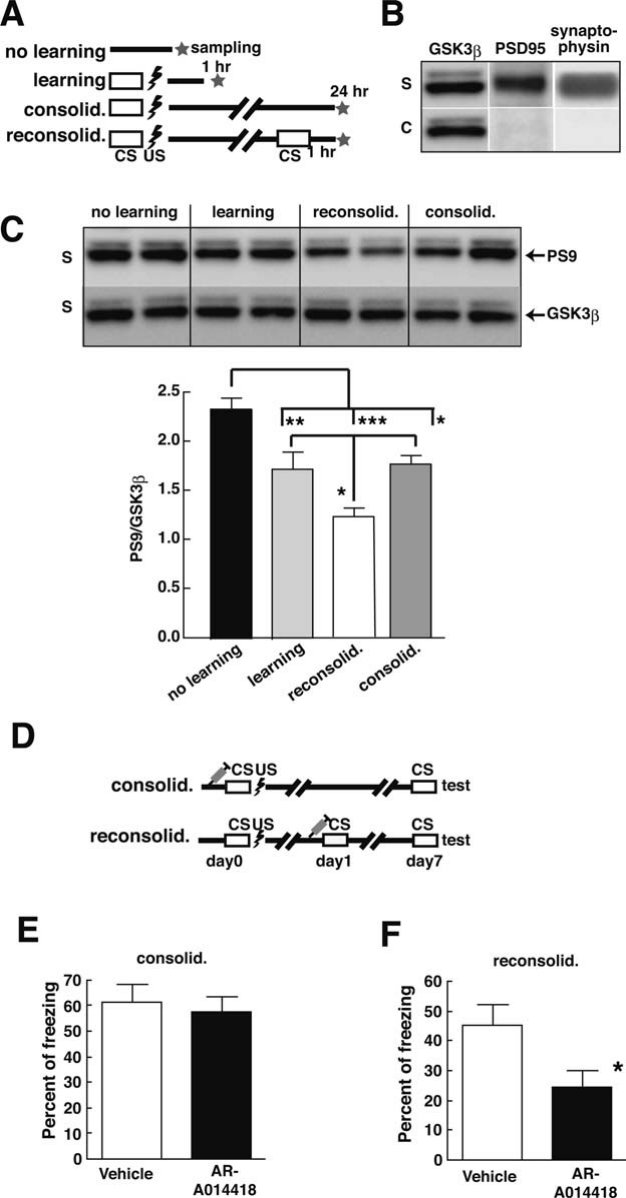
Memory reconsolidation requires GSK-3β activation. C57BL/6J mice (7 months old) received contextual fear conditioning according to the test schedule shown in A. Hippocampal GSK-3β levels were then assessed at different times during the testing. One and twenty-four hours after the presentation of paired conditioned and unconditioned stimuli (CS+US), mice were killed and phospho-Ser9 GSK-3β and total GSK-3β levels in hippocampal synaptic fractions were analyzed by Western blotting (B, C). GSK-3β was found in the synaptic (S) fraction (B). Mice showed reduced phospho-Ser9 GSK-3β levels after learning and during consolidation and reconsolidation compared to levels measured during “no learning” (C; upper panel). Ratios of phospho-Ser9 GSK-3β-to-total GSK-3β levels, which reflect the inhibition of GSK-3β activity, are shown in the histogram of panel of C. GSK-3β activity after learning (n = 5) and during consolidation (n = 5) was significantly increased compared to activity measured in the “no learning” group (n = 7). Further significant GSK-3β activation was seen in the reconsolidation group (n = 4). Statistical analysis was performed using the Tukey's multiple comparison test after one-way ANOVA; *, p<0.05; **, p<0.01; ***, p,0.001. Effects of a GSK-3 inhibitor on memory consolidation and reconsolidation (D–F). Times at which mice received an intraperitoneal injection of GSK-3 inhibitor and the task schedule for contextual fear conditioning are shown in D. Four groups of C57BL/6J mice were intraperitoneally injected with either GSK-3 inhibitor (AR-A014418; 30 mg/kg) or vehicle solution (approximately 100 µl of 10% DMSO/40% PEG solution for each animal) 1 h before consolidation (vehicle, n = 8; inhibitor, n = 8) or reconsolidation (vehicle, n = 8; inhibitor, n = 9). On day 7, memory performance was assessed by measuring freezing times under CS conditions. GSK-3 inhibitor administered before conditioning did not significantly affect memory performance on day 7 (E; p = 0.798, Mann-Whitney test). In contrast, when GSK-3 inhibitor was administered before reconsolidation, on day 7 freezing times of GSK-3 inhibitor-treated mice were significantly less than those of vehicle-treated mice (F; p = 0.036, Mann-Whitney test). Results are expressed as means±SEM; *, p<0.05.

To confirm the involvement of GSK-3β in memory reconsolidation, we first investigated GSK-3β activity in C57BL/6J mice during consolidation and reconsolidation in a CFC paradigm. Recently, GSK-3β in synapses have been reported to be involved in synaptic plasticity [20]. This prompted us to examine GSK-3β activity in synaptic fractions from mouse hippocampus relative to levels of phospho-Ser9 GSK-3β (Fig. 3B, C). One and twenty-four hours after CS+US presentation, phospho-Ser9 GSK-3β levels were reduced to about 72% of that of the control group, which did not receive the CS+US presentation, suggesting that GSK-3β was activated during memory consolidation. One hour after CS presentation during the reconsolidation step, phospho-Ser9 GSK-3β levels were further and significantly reduced to 50% of control levels (Fig. 3A, C), suggesting that GSK-3β might be activated during memory consolidation and an additional activation of GSK-3β might be required during the reconsolidation process.

If this additional GSK-3β activation is involved in memory reconsolidation, inhibition of GSK-3 might produce the same effects on memory reconsolidation as that observed in GSK^+/−^ mice. Thus, we next investigated the effect of GSK-3β inhibition on memory maintenance by intraperitoneal injection of the GSK-3 inhibitor AR-A014418 (30 mg/kg), followed by training on the CFC paradigm. To confirm inhibition of GSK-3, C57BL/6J mice were treated with the GSK-3 inhibitor before the consolidation step or before the reconsolidation step (1 h before CS+US presentation in the consolidation test, or 1 h before CS presentation in the reconsolidation test, respectively; Fig. 3D). The GSK-3 inhibitor reduced tau phosphorylation by 70% compared to the vehicle control, suggesting that the treatment inhibited GSK-3 activity (see Supporting information Fig. S6). Inhibiting GSK-3 before the consolidation step did not impair memory consolidation (Fig. 3E), but GSK-3 inhibition applied before the reconsolidation step significantly impaired memory reconsolidation (Fig. 3F). Thus, the memory reconsolidation step is more sensitive to GSK-3β activity than the memory consolidation step, and these results indicate that activation of GSK-3β might be required for memory reconsolidation.

## Discussion

The genetic reduction of GSK-3β and the pharmacological inhibition of GSK-3 impaired reconsolidation of hippocampus-dependent place memory, as demonstrated in the CFC test, a test that assesses hippocampus-mediated memory consolidation and reconsolidation. Hence, GSK-3β may be involved in memory reconsolidation in the hippocampus. The mechanism of memory formation and maintenance in the hippocampus, however, may also involve coordinated activity in other brain regions. Clearly, more work is needed to clarify the role of GSK-3β in cortex and other brain regions.

Although mice expressing the dominant negative form of GSK-3 display neuronal damage in striatum and cortex accompanied by impaired limb mobility [30], GSK^+/−^ mice did not display these phenomena. This discrepancy may due to GSK-3α activity. Although GSK^+/−^ reduces GSK-3β activity without affecting GSK-3α, dominant negative GSK-3 reduces both GSK-3α and GSK-3β activity in striatum and cortex. This reduction may cause neuronal vulnerability.

Both place learning in the MWM and context learning in the CFC paradigm reflect mainly hippocampal-dependent learning and memory. GSK^+/−^ mice showed retrograde amnesia in the MWM and CFC paradigms. Using the CFC paradigm, we demonstrated that GSK^+/−^, or pharmacological inhibition of GSK-3, impaired memory reconsolidation without affecting memory consolidation. Therefore, GSK^+/−^ mice—in which GSK-3β is genetically reduced—and WT mice treated with a GSK-3 inhibitor lose their ability to reconsolidate memory. Injecting a protein synthesis inhibitor (anisomycin) into dorsal hippocampus inhibits memory consolidation and reconsolidation in both place and context learning [31]–[33]. Thus, if GSK-3β is involved in a common mechanism of memory reconsolidation shared by both tasks, we would expect that the genetic reduction of GSK-3β to also impair memory reconsolidation of affected mice. Indeed, GSK^+/−^ mice did display impaired memory reconsolidation in both MWM and CFC tests.

During the first 3 days of training, GSK^+/−^ mice did not exhibit memory impairment (Fig. 1D). However, after 9 days of repetitive training, GSK^+/−^ mice exhibited retrograde amnesia (Fig. 1E). Artinian and colleagues [31] found that injecting anisomycin into CA3 after a single reactivation, inhibited memory reconsolidation following 4 sessions of three trials in one day in a place-learning version of the MWM paradigm. In our MWM training protocol, mice were repeatedly trained three trials everyday. The strength of memory in each day in our MWM protocol seems to be weaker than that in Artinians' protocol. The temporal dynamics of memory reconsolidation depend on the strength and age of the memory, such that younger and weaker memories are more easily reconsolidated than older and stronger ones [34]. Therefore, more than three days of repetitive training may be required for inhibiting memory reconsolidation by genetic reduction of GSK-3β (GSK^+/−^) under our MWM protocol.

One caveat in interpreting a retrograde amnesia effect in the CFC paradigm is the possibility of extinction of the conditioned response, because retrograde amnesia in repeated exposure to the same context without reinforcement can produce extinction. Therefore, it is possible that GSK-3β inhibition-induced retrograde amnesia may be due to facilitation of extinction or impairment of reconsolidation. However, the facilitation-of-extinction notion cannot explain the result showing that GSK^+/−^ mice displayed retrograde amnesia upon repetitive training in the MWM. Since maintenance of spatial memory in the MWM requires memory consolidation and reconsolidation [27] and since MWM training is performed under the existence of a reinforcer, spatial memory may not be affected by memory extinction. Therefore, retrograde amnesia related to GSK-3β inhibition could be due to an impairment of memory reconsolidation, not to facilitation of extinction.

NMDA-induced bidirectional synaptic plasticity (long term potentiation, LTP; long term depression, LTD) is believed to underlie memory formation [35]. During LTP, neurons store relevant information by synaptic tagging, which is thought to underlie a stable memory trace [36]. Recently, GSK-3β activation has been reported to be required for NMDA-dependent LTD induction [20]. Furthermore, overexpression of GSK-3β inhibits LTP induction [37]. Therefore, GSK-3β could modulate the balancing of LTP and LTD in an activity-dependent manner. Overexpression of GSK-3β impairs acquisition of reference memory in a novel object recognition task [38]. As we have shown in this report, the inhibition of GSK-3β blocks memory reconsolidation but not memory acquisition or consolidation. These observations suggest that activated GSK-3β may contribute to memory reconsolidation by controlling LTP/LTD balance.

If LTP and LTD contribute to the formation and maintenance of memory traces, memory acquisition and consolidation processes may preferentially depend on LTP. In the case of memory reconsolidation, LTD may be more important for maintaining a prior potentiated circuit by competitive synaptic maintenance [39]. GSK-3β activation may help protect a stable memory trace from corruption through additional synaptic connections or changes during recall and reconsolidation. Although this idea may be supported by the observation that greater activation of GSK-3β is required in the reconsolidation process rather than in the consolidation process, more precise mechanisms need to be clarified.

GSK-3β is involved in NFT formation in AD. During normal aging, NFTs are observed in the entorhinal cortex. This may represent a very early pathological change of sporadic AD. Because memory reconsolidation is required for GSK-3β activation, tau might be hyperphosphorylated when memory is reconsolidated. As we recently reported, the accumulation of hyperphosphorylated tau impairs learning and memory during old age [40]. Therefore, frequent activation of memory reconsolidation in older brains may damage learning and memory through the hyperphosphorylation of tau. Thus, GSK-3β does have an important role in memory maintenance in the adult brain, but it may also cause NFT formation in the aged brain.

## Materials and Methods

### Animals

Male C57/BL6J mice aged ∼7 months were used for all experiments. Male GSK^+/−^ mice (7–14 months) were maintained by backcrossing with C57/BL6J mice. Mice were individually housed and kept on a 12-h light/dark schedule. All mice had free access to food and water. To assess GSK-3β activation during memory consolidation and reconsolidation, we subjected C57BL/6J mice to a CFC paradigm. At the indicated time points shown in Fig. 3a, mice were killed by cervical dislocation and their hippocampi were removed and prepared for biochemical analysis of GSK-3β. All experiments were performed according to procedures approved by the Animal Experiments Committee of the Institute of Physical and Chemical Research (RIKEN).

### Antibodies

We used the following antibodies: rabbit polyclonal anti-GSK-3, mouse monoclonal anti-GSK-3β (Transduction Lab, Inc.); rabbit polyclonal anti-phospho-Ser9 GSK-3β (Cell Signaling Technology, Inc.).

### Western blotting

Mouse hippocampi were homogenized in Tris-buffered saline (TBS; 10 mM Tris, 150 mM NaCl [pH 7.4]) containing protease inhibitors (1 µg/ml antipine, 5 µg/ml pepstatin, 5 µg/ml leupeptin, 2 µg/ml aprotinin, and 0.5 µM 4-(2-aminoethyl)benzenesulfonyl fluoride hydrochloride) and phosphatase inhibitors (1 mM NaF, 0.4 mM Na_3_VO_4_, and 0.5 mM okadaic acid). After centrifugation at 27,000 *g* for 20 min, the supernatant was collected. TBS-soluble materials were solubilized in Laemmli sample buffer and subjected to SDS-PAGE. Next, we purified synaptic fractions from hippocampus. Hippocampi from mouse brains were homogenized in 8 volumes of 10 mM HEPES buffer containing protease inhibitors and phosphatase inhibitors, 5 mM EDTA, and 300 mM sucrose, and centrifuged at 12,000 *g* for 10 min. The supernatant was centrifuged at 39,800 *g* for 40 min, and the nuclear fraction was completely removed. The synaptic fraction was purified from the supernatant by centrifugation at 120,000 *g* for 40 min. The cytoplasmic fraction was obtained by centrifugation at 260,000 *g* for 2 h. Synaptic and cytoplasmic fractions were subjected to SDS-PAGE, and separated proteins were blotted onto nitrocellulose membranes (Schleicher & Schuell Bioscience). The membranes were then incubated with primary antibody, followed by the species-appropriate HRP-conjugated secondary antibody. Chemiluminescent detection (ECL; GE Healthcare Bio Science) was used for visualization. Quantitation and visual analysis of immunoreactivity were performed with a computer-linked LAS-3000 Bio-Imaging Analyzer System (Fujifilm).

### Morris water maze test

To assess place learning and memory performance of mice, we used a cylindrical test apparatus (1 m in diameter) and task closely fashioned after the Morris water maze according to our previous report [35]. To assess learning, for each mouse we calculated the distance between the mouse and the platform every 0.5 sec until the mouse reached the platform. Next, we calculated the total distance traveled by the mouse as it searched for the platform. This error score was used as a measure of learning performance. Lower scores indicated better learning and memory. Each mouse was subjected to three learning trials per day for nine successive days. A single probe test was given on the tenth day, in which the platform was removed from the maze in the mouse's absence. The mouse was introduced into the maze as before and allowed to search for the missing platform for 60 sec. The percentage of time the mouse spent searching in each quadrant was used as an index of memory performance. Statistical analyses were conducted using PRISM4 (GraphPad Software Inc.). Data were analyzed using the Friedman test or two-way ANOVA, unless noted otherwise.

### Contextual fear-conditioning (CFC) test for memory consolidation and reconsolidation

To test the effect of GSK-3β reduction on memory consolidation and reconsolidation, GSK^+/−^ mice and GSK-3 inhibitor-treated C57BL/6J mice were randomly selected and assessed on a CFC test. The test apparatus consisted of a 60 cm×40 cm×40 cm sound-proof box containing a 10 cm×10 cm×10 cm transparent plastic chamber with a steel grid floor (for delivering electrical foot shocks) and a CCD camera. To examine memory consolidation, we exposed individual to the novel environment (plastic chamber) for 5 min (conditioning stimulus, CS), and then delivered three sequential foot shocks (unconditioning stimulus, US; shock intensity = 0.5 mV; duration = 2 sec; inter-stimuli interval = 60 sec). Seven days after CS presentation, conditioned animals were re-exposed to the same plastic chamber for 4 min, and freezing time was measured with custom software based on Matlab (version 7.2, Mathworks Co. Ltd.). To examine memory reconsolidation, we exposed mice to the environmental CS for 5 min. One day after CS presentation, conditioned animals were exposed to the same plastic chamber for 4 min, and freezing time was monitored as before. Six days after first being exposed to the same plastic chamber, mice were re-exposed to the chamber, and memory reconsolidation was assessed by measuring freezing time. The same behavioral analyses were applied in experiments testing the affect of GSK-3 inhibition on C57BL/6J mice and biochemical analysis of GSK-3β activity during contextual fear conditioning.

## Supporting Information

Figure S1 Generation of GSK-3β heterozygote mouse. Using homologous recombination, we flanked the exon-encoding catalytic domain of GSK-3β with loxP elements. Floxed GSK-3β mice were crossed with mice expressing Cre recombinase under the control of the EIIa promoter, and their progeny were crossed with C57/BL6J mice (A). The offspring of this latter cross underwent PCR tail DNA analysis using the primer sets indicated, and mice harboring the delta allele were selected (B). The brains of six wild-type (GSK-3β+/+; WT littermate) and six heterozygous (GSK-3β+/−; GSK+/−) mice were homogenized and GSK-3α and GSK-3β expression levels were examined by Western blotting (C). GSK-3β (D) and GSK-3{lwoer case alpha} (F) expression levels were quantified with a computer-linked LAS-3000 Bio-Imaging Analyzer System. Although there were no significant differences in the GSK-3α expression levels of WT and GSK+/− mice (n = 6, p = 0.1797, Mann-Whitney test), the GSK-3β expression levels of GSK+/− mice were about 50% of that of the WT littermates (n = 6, p = 0.0022, Mann-Whitney test). Reduced GSK-3β activity in GSK+/− mice was confirmed by incorporating radiolabeled 32P into GSK-3 substrate peptide (E) (n = 6, p = 0.0022, Mann-Whitney test). Results are expressed as means±SEM; *, p<0.05; **, p<0.01; ***, p<0.001. The total amount (C, E) and relative activity (F) of GSK-3β in GSK+/− mice were approximately 50% and 70%, respectively, of those in WT littermates. The total amount and relative activity of GSK-3α, a homologue of GSK-3β, in GSK+/− mice were similar to those in WT mice (C, D). Consistent with a previously report [20], our GSK+/− mice were healthy, fertile, and showed no changes in circadian rhythm, life span, motor control, and locomotor activity compared to WT mice (see Supporting information Fig. S2). GSK+/− mice, however, displayed reduced GSK-3β activity, without showing GSK-3α compensation.
(0.13 MB PDF)

Figure S2 Basic characteristics (circadian rhythm, locomotor activity, motor control activity, and life span) of GSK+/− mice. GSK+/− mice showed similar awake/rest patterns as WT littermate mice when housed under 12-h light/dark conditions (A). After measuring the locomotor activity of three GSK+/− and three WT mice for 3 successive days under home-cage conditions, we found that the total daily locomotor activity (number of beam crossings) of GSK+/− mice during circadian monitoring did not significantly differ from that of WT littermate mice (B). Motor control activity (C) was tested using an accelerated rotarod test (1.5–15 rpm/3 min). GSK+/− and WT littermate mice did not show significant motor-skill differences (Mann-Whitney test). Furthermore, GSK+/− and WT littermate mice showed comparable survival times (D) (Log-rank test, p>0.05).
(0.11 MB PDF)

Figure S3 Path length (A) and latency (B) of WT and GSK+/− mice in the MWM during 9 days of training. During the first 3 days of training, both WT and GSK+/− mice showed reduced latency to platform and shorter path-length to platform after repetitive training. After longer, subsequent training, WT mice showed reduced latency to platform and shorter path-length to platform, but GSK+/− mice did not. This tendency is similarly reflected in the error score plot of Fig. 1B. Because the error score is a value representing the cumulative distance a mouse travels to find the platform during each trial, the error score not only reflects a mouse's path-length information during training but also it also reflects a mouse's trace information until it reaches the platform.
(0.08 MB PDF)

Figure S4 The emotional responses of GSK+/− mice to the Morris water maze were determined by assessing swim speed (A) and thigmotaxis tendency (B). In this analysis, we used only data obtained from the first training day to omit the influence of learning. Both emotional parameters indicated that no significant differences exist between GSK+/− (n = 19) and WT littermate (n = 20) mice (p>0.05, Mann-Whitney test).
(0.05 MB PDF)

Figure S5 The effect of aging on place memory formation was investigated by comparing probe test scores (percentage of stay time in the target quadrant) after 9 days of training (Fig. 1E). Three age groups (7–9 months, 10–11 months, 12–14 months) of WT and GSK+/− mice were assessed. Although the relatively wide range of ages, 7–14 months, did not affect place memory formation (two-way ANOVA analysis; aging factor, F = 0.4772,p = 14.86), genotype, WT vs. GSK+/−, did formation (two-way ANOVA analysis; genotype factor, F = 6.020,p = 0.0196).
(0.08 MB PDF)

Figure S6 AR-A014418 inhibited tau phosphorylation, as shown in the Western blots probed with TauC, a phosphorylation independent anti-tau antibody; PHF1 (generously provided by Dr. Peter Davies, Albert Einstein College of Medicine, NY), a phosphorylation-dependent anti-tau antibody; and Tau1 (CHEMICON, Temecula, CA), a non-phosphorylation-dependent anti-tau antibody, which recognizes non-phosphorylated Ser199 and Ser202. AR-A014418 reduced tau phosphorylation (PHF1 immunoreactivity was reduced, Tau1 immunoreactivity was increased, TauC immunoreactivity was unchanged) to 70% of that produced by vehicle injection alone, suggesting that peripheral treatment with a GSK-3 inhibitor inhibited GSK-3 activity by 30%.
(0.08 MB PDF)
