# Supplementary figures and images for: GSK-3β Is Required for Memory Reconsolidation in Adult Brain
Source: PLoS One. 2008 Oct 28;3(10):e3540. doi: 10.1371/journal.pone.0003540 (PMC2568810; doi:10.1371/journal.pone.0003540)

**A**

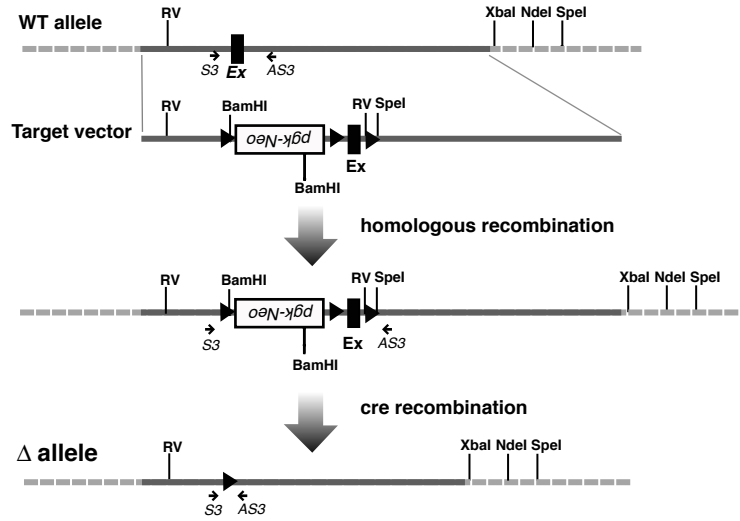

**B**

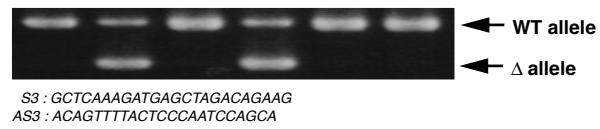

**C**

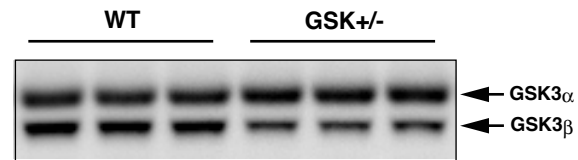

**D**

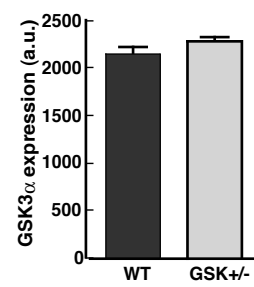

**E**

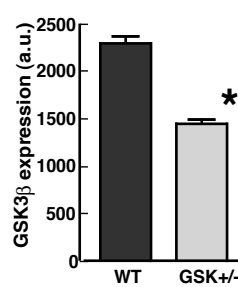

**F**

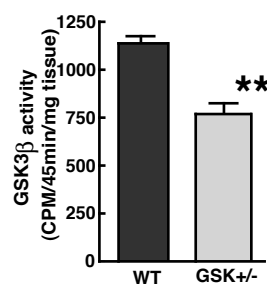

Supplement: Figure S1 — Generation of GSK-3β heterozygote mouse. Using homologous recombination, we flanked the exon-encoding catalytic domain of GSK-3β with loxP elements. Floxed GSK-3β mice were crossed with mice expressing Cre recombinase under the control of the EIIa promoter, and their progeny were crossed with C57/BL6J mice (A). The offspring of this latter cross underwent PCR tail DNA analysis using the primer sets indicated, and mice harboring the delta allele were selected (B). The brains of six wild-type (GSK-3β+/+; WT littermate) and six heterozygous (GSK-3β+/−; GSK+/−) mice were homogenized and GSK-3α and GSK-3β expression levels were examined by Western blotting (C). GSK-3β (D) and GSK-3{lwoer case alpha} (F) expression levels were quantified with a computer-linked LAS-3000 Bio-Imaging Analyzer System. Although there were no significant differences in the GSK-3α expression levels of WT and GSK+/− mice (n = 6, p = 0.1797, Mann-Whitney test), the GSK-3β expression levels of GSK+/− mice were about 50% of that of the WT littermates (n = 6, p = 0.0022, Mann-Whitney test). Reduced GSK-3β activity in GSK+/− mice was confirmed by incorporating radiolabeled 32P into GSK-3 substrate peptide (E) (n = 6, p = 0.0022, Mann-Whitney test). Results are expressed as means±SEM; *, p<0.05; **, p<0.01; ***, p<0.001. The total amount (C, E) and relative activity (F) of GSK-3β in GSK+/− mice were approximately 50% and 70%, respectively, of those in WT littermates. The total amount and relative activity of GSK-3α, a homologue of GSK-3β, in GSK+/− mice were similar to those in WT mice (C, D). Consistent with a previously report [20], our GSK+/− mice were healthy, fertile, and showed no changes in circadian rhythm, life span, motor control, and locomotor activity compared to WT mice (see Supporting information Fig. S2). GSK+/− mice, however, displayed reduced GSK-3β activity, without showing GSK-3α compensation. (0.13 MB PDF) [file pone.0003540.s001.pdf]

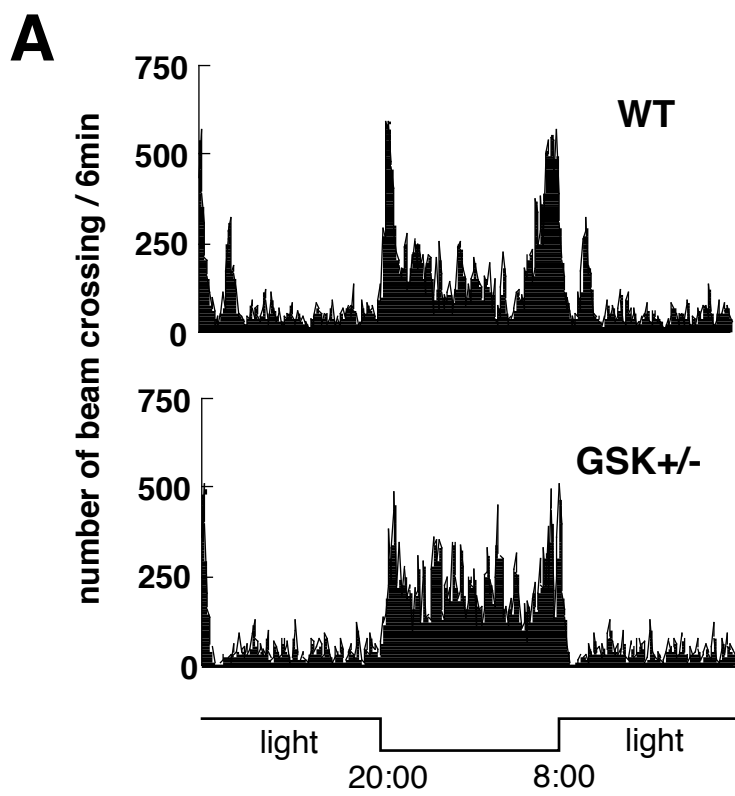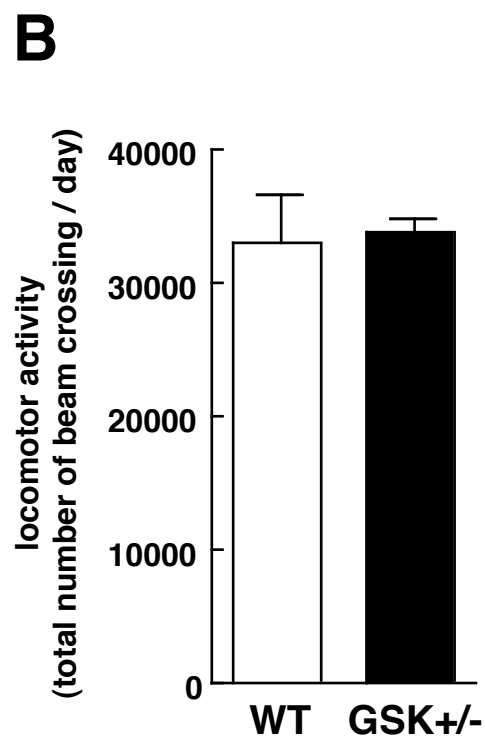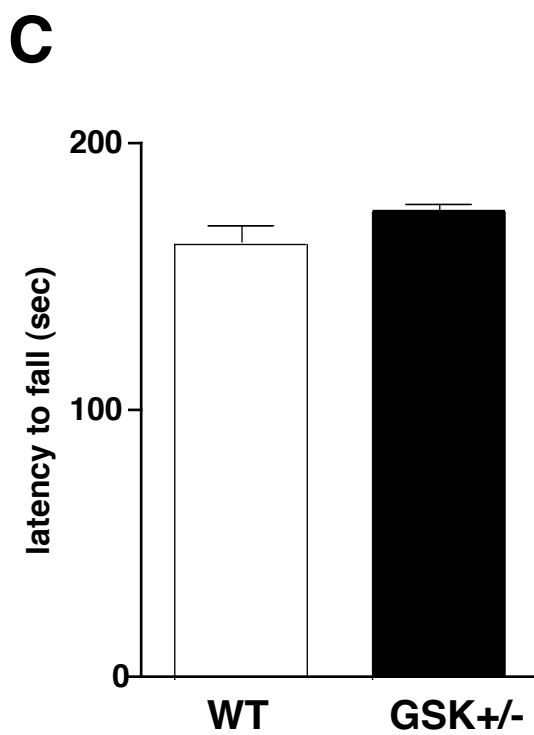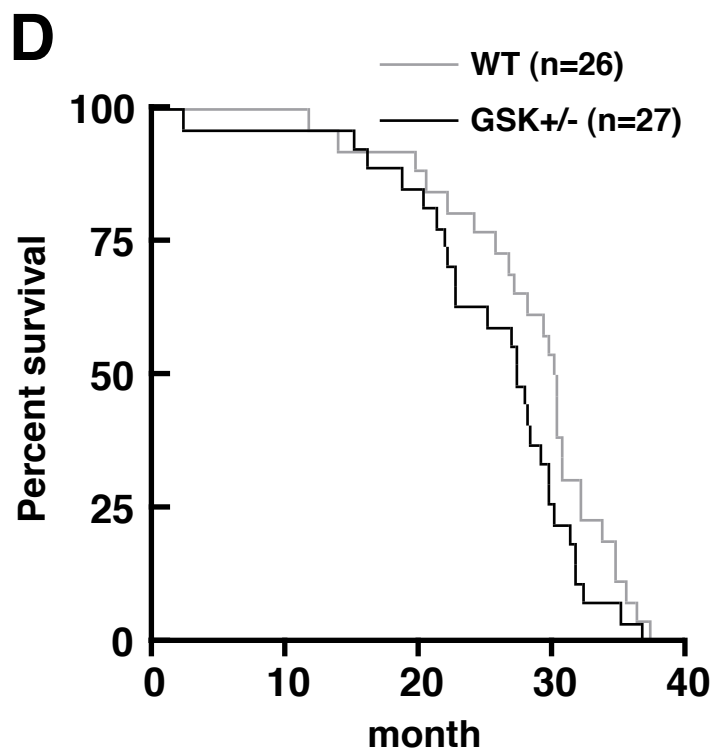

Supplement: Figure S2 — Basic characteristics (circadian rhythm, locomotor activity, motor control activity, and life span) of GSK+/− mice. GSK+/− mice showed similar awake/rest patterns as WT littermate mice when housed under 12-h light/dark conditions (A). After measuring the locomotor activity of three GSK+/− and three WT mice for 3 successive days under home-cage conditions, we found that the total daily locomotor activity (number of beam crossings) of GSK+/− mice during circadian monitoring did not significantly differ from that of WT littermate mice (B). Motor control activity (C) was tested using an accelerated rotarod test (1.5–15 rpm/3 min). GSK+/− and WT littermate mice did not show significant motor-skill differences (Mann-Whitney test). Furthermore, GSK+/− and WT littermate mice showed comparable survival times (D) (Log-rank test, p>0.05). (0.11 MB PDF) [file pone.0003540.s002.pdf]

**A**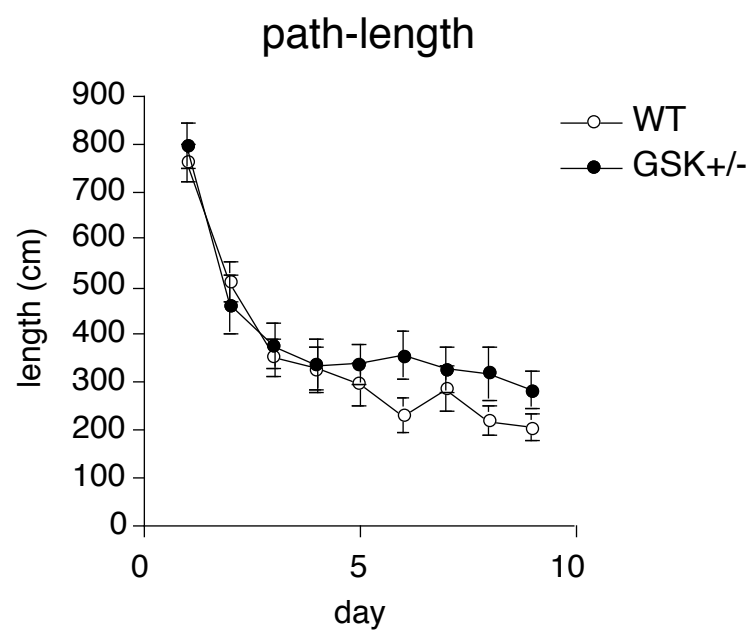**B**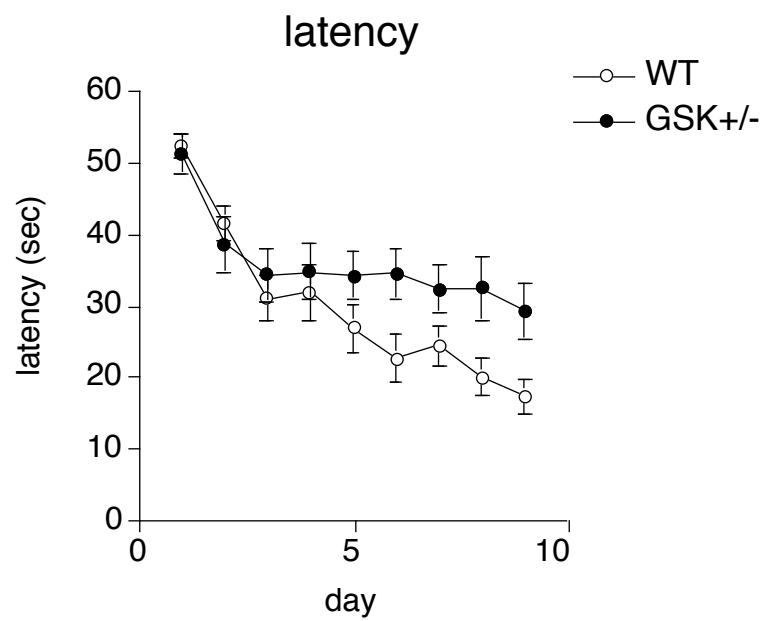

Supplement: Figure S3 — Path length (A) and latency (B) of WT and GSK+/− mice in the MWM during 9 days of training. During the first 3 days of training, both WT and GSK+/− mice showed reduced latency to platform and shorter path-length to platform after repetitive training. After longer, subsequent training, WT mice showed reduced latency to platform and shorter path-length to platform, but GSK+/− mice did not. This tendency is similarly reflected in the error score plot of Fig. 1B. Because the error score is a value representing the cumulative distance a mouse travels to find the platform during each trial, the error score not only reflects a mouse's path-length information during training but also it also reflects a mouse's trace information until it reaches the platform. (0.08 MB PDF) [file pone.0003540.s003.pdf]

**A**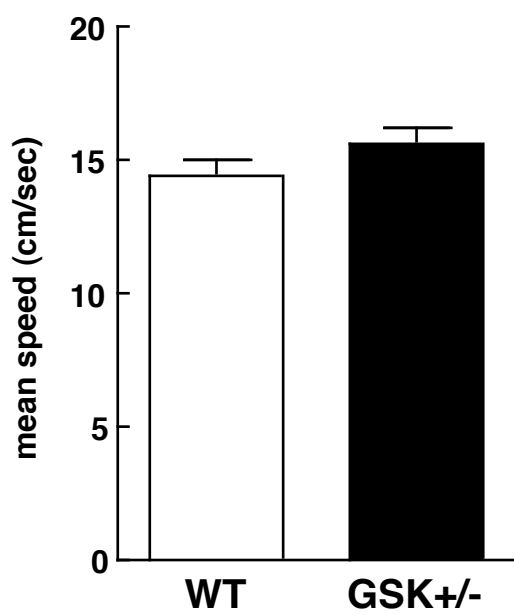**B**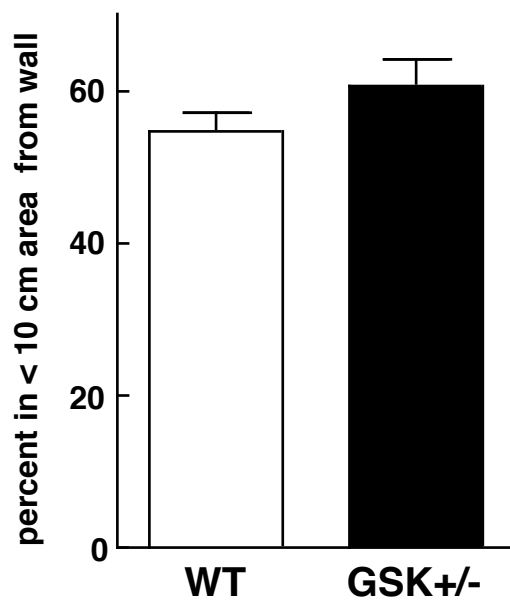

Supplement: Figure S4 — The emotional responses of GSK+/− mice to the Morris water maze were determined by assessing swim speed (A) and thigmotaxis tendency (B). In this analysis, we used only data obtained from the first training day to omit the influence of learning. Both emotional parameters indicated that no significant differences exist between GSK+/− (n = 19) and WT littermate (n = 20) mice (p>0.05, Mann-Whitney test). (0.05 MB PDF) [file pone.0003540.s004.pdf]

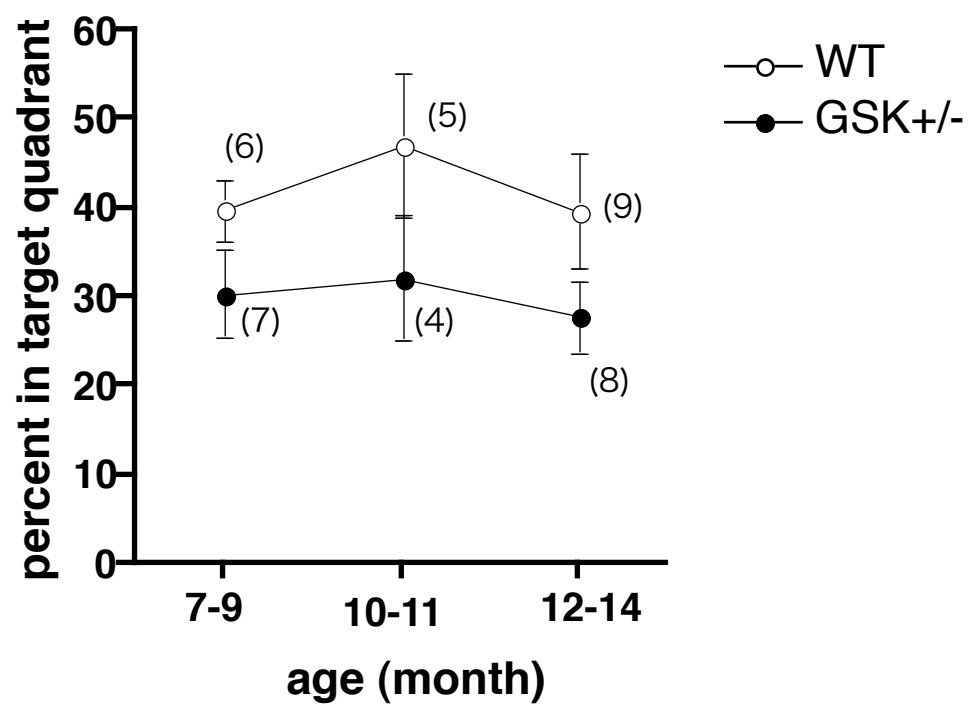

Supplement: Figure S5 — The effect of aging on place memory formation was investigated by comparing probe test scores (percentage of stay time in the target quadrant) after 9 days of training (Fig. 1E). Three age groups (7–9 months, 10–11 months, 12–14 months) of WT and GSK+/− mice were assessed. Although the relatively wide range of ages, 7–14 months, did not affect place memory formation (two-way ANOVA analysis; aging factor, F = 0.4772,p = 14.86), genotype, WT vs. GSK+/−, did formation (two-way ANOVA analysis; genotype factor, F = 6.020,p = 0.0196). (0.08 MB PDF) [file pone.0003540.s005.pdf]

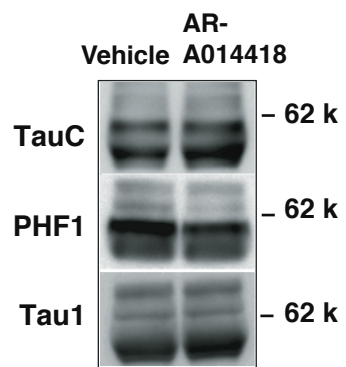

Supplement: Figure S6 — AR-A014418 inhibited tau phosphorylation, as shown in the Western blots probed with TauC, a phosphorylation independent anti-tau antibody; PHF1 (generously provided by Dr. Peter Davies, Albert Einstein College of Medicine, NY), a phosphorylation-dependent anti-tau antibody; and Tau1 (CHEMICON, Temecula, CA), a non-phosphorylation-dependent anti-tau antibody, which recognizes non-phosphorylated Ser199 and Ser202. AR-A014418 reduced tau phosphorylation (PHF1 immunoreactivity was reduced, Tau1 immunoreactivity was increased, TauC immunoreactivity was unchanged) to 70% of that produced by vehicle injection alone, suggesting that peripheral treatment with a GSK-3 inhibitor inhibited GSK-3 activity by 30%. (0.08 MB PDF) [file pone.0003540.s006.pdf]
